# *CCNE1* and *E2F1* Partially Suppress G1 Phase Arrest Caused by Spliceostatin A Treatment

**DOI:** 10.3390/ijms222111623

**Published:** 2021-10-27

**Authors:** Kei Kikuchi, Daisuke Kaida

**Affiliations:** 1 School of Medicine, University of Toyama, Toyama 930-0194, Japan; s1650031@ems.u-toyama.ac.jp; 2 Faculty of Medicine, Academic Assembly, University of Toyama, Toyama 930-0194, Japan

**Keywords:** pre-mRNA splicing, spliceostatin A, cell cycle, G1 phase, cyclin E, E2F1

## Abstract

The potent splicing inhibitor spliceostatin A (SSA) inhibits cell cycle progression at the G1 and G2/M phases. We previously reported that upregulation of the p27 cyclin-dependent kinase inhibitor encoded by *CDKN1B* and its C-terminal truncated form, namely p27*, which is translated from *CDKN1B* pre-mRNA, is one of the causes of G1 phase arrest caused by SSA treatment. However, the detailed molecular mechanism underlying G1 phase arrest caused by SSA treatment remains to be elucidated. In this study, we found that SSA treatment caused the downregulation of cell cycle regulators, including *CCNE1*, *CCNE2*, and *E2F1*, at both the mRNA and protein levels. We also found that transcription elongation of the genes was deficient in SSA-treated cells. The overexpression of *CCNE1* and *E2F1* in combination with *CDKN1B* knockout partially suppressed G1 phase arrest caused by SSA treatment. These results suggest that the downregulation of *CCNE1* and *E2F1* contribute to the G1 phase arrest induced by SSA treatment, although they do not exclude the involvement of other factors in SSA-induced G1 phase arrest.

## 1. Introduction

Cell cycle progression is closely controlled by cell cycle regulators. Among these regulators, cyclin family proteins and cyclin-dependent kinases (CDKs) promote cell cycle progression [1,2]. In G1/S phase transition, cyclin E1 and cyclin E2 (collectively known as cyclin E), along with Cdk2, are the key players that phosphorylate a variety of substrates [3,4,5,6]. Rb is a substrate of the cyclin E–Cdk2 complex; it binds the E2F1 transcription factor and represses the transcriptional activity of E2F1 [7,8]. After phosphorylation by cyclin E–Cdk2, Rb releases E2F1, and E2F1, in turn, activates the transcription of numerous genes that drive the initiation of the S phase. The genes encoding cyclin E are controlled by E2F1, and thus the cyclin E–Cdk2 complex and E2F1 form a positive feedback loop that promotes G1/S phase transition [3,4,6,9]. The cyclin E–Cdk2 complex also phosphorylates Smad3, CBP/p300, and NPAT, leading to cell cycle progression [10,11,12,13]. Conversely, CDK inhibitors repress cell cycle progression by inhibiting the kinase activity of CDKs [2,9]. p27 is one of the CDK inhibitors that control G1/S phase transition [9,14,15]. It binds to and inhibits the cyclin E–Cdk2 complex to block cell cycle progression at the G1 phase. The cyclin E–Cdk2 complex negatively regulates p27 through phosphorylation of p27, which induces ubiquitination and proteasomal degradation of p27 [16,17,18]. Collectively, these factors regulate G1/S phase transition in a coordinated manner.

In higher eukaryotes, most precursor mRNAs (pre-mRNAs) consist of exon sequences and intervening sequences (introns) [19,20]. During mRNA processing, introns are excised and exons are joined by the mRNA splicing machinery to form mature mRNA. Because mature mRNA serves as the template for translation, defects in mRNA splicing can result in the downregulation of functional proteins and/or the production of abnormal proteins translated from pre-mRNA. We previously reported that a truncated form of the p27 CDK inhibitor, namely p27*, is produced in cells treated with the splicing inhibitor spliceostatin A (SSA) via translation from pre-mRNA [21]. Furthermore, we found that splicing inhibition by SSA causes cell cycle arrest at the G1 and G2/M phases, and the expressions of p27 and p27* are upregulated in G1-arrested cells [21,22,23]. These results prompted us to investigate whether upregulation of p27 and p27* expression is the reason for G1 phase arrest induced by splicing inhibition; we found that upregulation of p27 and p27* expression causes G1 phase arrest but knockdown of p27 and p27* is not sufficient for complete suppression of SSA-induced G1 phase arrest [23]. Therefore, other factors besides p27 and p27* appear to contribute to G1 phase arrest caused by splicing inhibition. Notably, SSA has very potent antitumor activity, and cell cycle arrest is thought to be a cause of this activity [21]. Therefore, identification of the additional factors involved in SSA-mediated induction of G1 phase arrest will deepen our understanding of the molecular mechanism underlying the interconnection between splicing and cell cycle arrest as well as facilitate the development of novel cancer therapies based on splicing inhibitors. In this study, we sought to identify the additional factors that contribute to G1 phase arrest induced by SSA and to investigate the molecular mechanism of splicing inhibition-induced cell cycle arrest.

## 2. Results

### 2.1. SSA Treatment Decreases Cyclin E1, Cyclin E2, and E2F1 Levels

**Figure 1:**
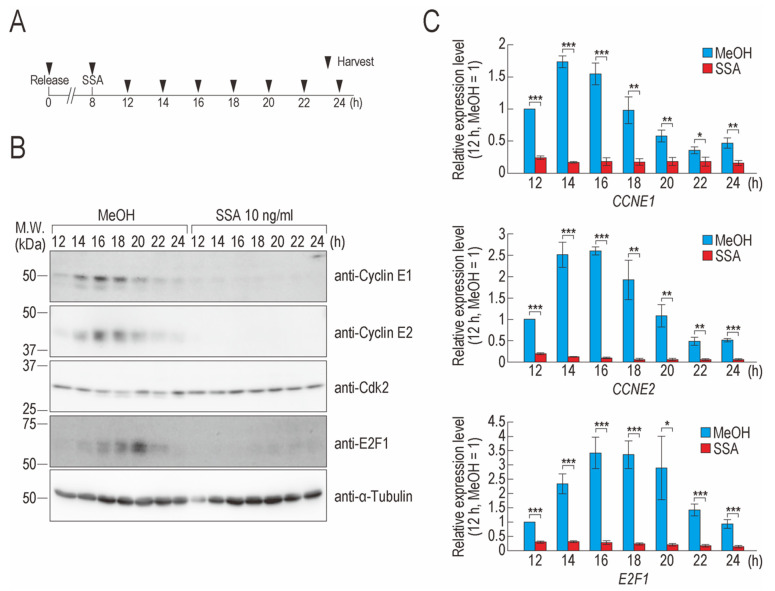
SSA treatment decreases the expressions of cell cycle regulators. (**A**) Eight hours after release from a double thymidine block, synchronized HeLa S3 cells were treated with MeOH or 10 ng/mL SSA. The cells were then harvested at the indicated time points (black triangles). (**B**) The protein levels of cell cycle regulators were analyzed using immunoblotting. The protein level of α-tubulin was measured as an internal loading control. (**C**) The relative expression levels of *CCNE1*, *CCNE2*, and *E2F1* were analyzed using qRT-PCR. Error bars indicate standard deviation (n = 3). Statistical significance was determined using a two-tailed *t*-test (* *p* < 0.05; ** *p* < 0.01; *** *p* < 0.001).

We previously reported that the upregulation of p27 and p27* expression is a cause of G1 phase arrest in SSA-treated cells [21,23]. However, because knockdown of p27 and p27* was insufficient for complete suppression of SSA-induced G1 phase arrest [23], we presumed that another mechanism of G1 phase arrest must be affected by SSA treatment. To examine the entire molecular mechanism underlying G1 phase arrest induced by SSA, we synchronized the cell cycle of HeLa S3 cells using thymidine and performed time-course analyses of cyclin E1, cyclin E2, E2F1, and Cdk2 protein levels, which are key factors for G1/S phase transition [1,4,24]. In MeOH-treated cells, cyclin E1, cyclin E2, and E2F1 started to accumulate at 14 h, after which their levels decreased and were hardly detected at 24 h. In contrast, Cdk2 was observed at all time points in MeOH-treated cells (Figure 1A,B). In SSA-treated cells, cyclin E1, cyclin E2, and E2F1 were barely detected. While the protein level of Cdk2 was not affected by SSA treatment, two bands of Cdk2 were observed at 8 h (Figure 1A,B and Figure S1). The phosphorylation of Cdk2 is regulated in a cell cycle-dependent fashion and Cdk2 is partially dephosphorylated at S and G2 phases [25]; therefore, some of the cells at 8 h appeared to be in the S or G2 phases.

We next measured the mRNA levels of *CCNE1*, *CCNE2*, and *E2F1* to determine whether they also decreased following SSA treatment. The levels of these mRNAs in SSA-treated cells were significantly lower than those in control cells at all time points (Figure 1C). These results suggest that splicing inhibition leads to a decrease in the mRNA levels of cyclin E1, cyclin E2, and E2F1, which leads to decreased protein levels.

### 2.2. Transcription Elongation of CCNE1, CCNE2, and E2F1 Is Defective in SSA-Treated Cells

**Figure 2:**
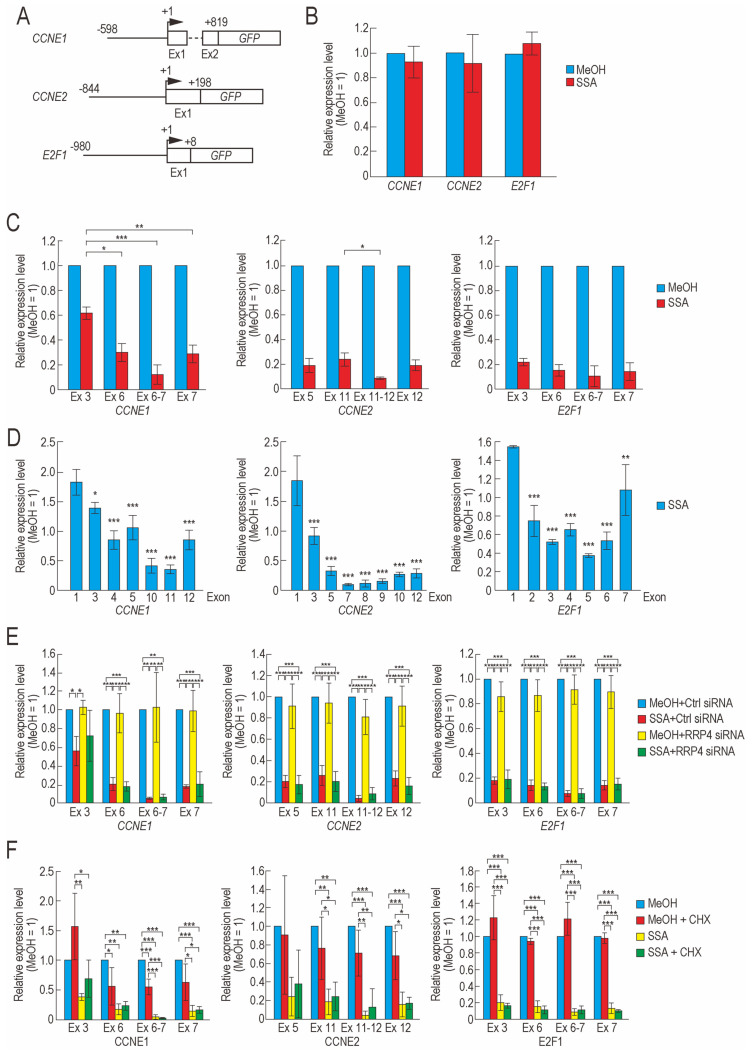
SSA inhibits transcription elongation of cell cycle regulator genes. (**A**) Schematics of *CCNE1* promoter-GFP, *CCNE2* promoter-GFP, and *E2F1* promoter-GFP reporter genes. Boxes, dotted horizontal lines, solid horizontal lines, and arrowheads represent exons, introns, promoter regions, and transcription start sites, respectively. (**B**) Synchronized cells were released from the first thymidine block and transfected with reporter plasmids. Eight hours after release from the second thymidine block, the cells were treated with 10 ng/mL SSA or MeOH, and newly synthesized RNAs were labeled with EU between 4 and 5 h after SSA treatment. Labeled RNAs were then analyzed using qRT-PCR. (**C**) Eight hours after release from the second thymidine block, SSA treatment and nascent RNA labeling were performed as described in (**B**). Labeled RNAs were analyzed using qRT-PCR. (**D**) Reanalysis of our previous exon array data [27]. Each bar represents the relative expression level of exons. (**E**) Synchronized cells were released from the first thymidine block and transfected with *RRP4* siRNA (RRP) or control siRNA (Ctrl). Eight hours after release from the second thymidine block, SSA treatment and nascent RNA labeling were performed as described in (**B**). Labeled RNAs were analyzed using qRT-PCR. (**F**) Eight hours after release from the double thymidine block, the cells were treated with 10 ng/mL SSA and 10 μg/mL CHX, and nascent RNA labeling was performed as described in (**B**). Labeled RNAs were analyzed using qRT-PCR. Error bars indicate standard deviation (n = 3). Statistical significance was investigated using a two-tailed *t*-test (**B**), one-way ANOVA, and Tukey’s test (**C**,**E**,**F**) or one-way ANOVA and Dunnett’s test (**D**) (* *p* < 0.05; ** *p* < 0.01; *** *p* < 0.001).

We investigated the decrease in the mRNA levels of *CCNE1*, *CCNE2*, and *E2F1* in SSA-treated cells. One possible explanation for this observation is that the levels of transcription factors important for the transcription initiation of the three genes are decreased, leading to a decrease in the mRNA levels of *CCNE1*, *CCNE2*, and *E2F1*. To test this hypothesis, we constructed three reporter genes containing the *GFP* gene under the control of a promoter region from *CCNE1*, *CCNE2*, or *E2F1* (Figure 2A). We measured the level of GFP mRNA in cells transfected with the reporters and found that SSA treatment did not affect the level of GFP mRNA in all three cases (Figure 2B). It is important to note that we analyzed only newly transcribed mRNA to detect the effect of SSA on transcription initiation and to minimize the effect of mRNA degradation. This result suggests that transcription initiation of *CCNE1*, *CCNE2*, and *E2F1* was not defective in SSA-treated cells.

Another possibility is that SSA inhibits the transcription elongation of the genes. We previously reported that transcription elongation of at least ~20% of genes is defective in SSA-treated cells [26,27,28]. Therefore, SSA may inhibit the transcription elongation of these three genes, which in turn decreases their expression. To test this hypothesis, we purified newly synthesized mRNA from cells after SSA treatment and measured the levels of some exons or exon junctions within these genes (Figure 2C). The relative expression levels of the exons and exon junctions decreased following SSA treatment (Figure 2C). The relative expression levels of exons 6 and 7 of *CCNE1* were much lower than those of exon 3 of *CCNE1*, indicating that SSA treatment causes a transcription elongation defect between exon 3 and 6 of *CCNE1* (Figure 2C). However, in *CCNE2* and *E2F1*, the relative expression levels of upstream exons (i.e., *CCNE2* Ex5 and *E2F1* Ex3) were almost identical to those of downstream exons (i.e., *CCNE2* Ex11 and 12 and *E2F1* Ex6 and 7), which suggests that SSA did not cause a transcription elongation defect between *CCNE2* Ex5 and Ex11 or between *E2F1* Ex3 and Ex6. We speculate that the transcription elongation defect occurs at positions further upstream of *CCNE2* exon 5 and *E2F1* exon 3.

To measure the levels of upstream exons using quantitative reverse transcription PCR (qRT-PCR), we designed primer sets for upstream exons; however, these primers did not work well because some upstream exons are GC-rich, whereas others are too short to design both forward and reverse primers for one exon. Therefore, to measure the levels of upstream exons, we reanalyzed the exon array data that we had previously published, in which we analyzed only newly synthesized mRNA to detect the effect of SSA on transcription [27]. We found that the relative expression levels of exon 1 of *CCNE2* and *E2F1* were significantly higher than those of the downstream exons (Figure 2D), which suggested that a transcription elongation defect occurs at upstream regions in *CCNE2* and *E2F1*. Notably, downregulation of the downstream exons did not appear to be caused by genome-wide mRNA degradation from the 3′ end to 5′ end during the sample preparation, as we previously reported that ~20% of genes show downregulation at the 3′ end while ~80% did not show downregulation [27]. Together, these findings indicate that the expression levels of all three genes were decreased in SSA-treated cells from a transcription elongation defect.

In addition to the transcription elongation defect, pre-mRNA degradation might contribute to the reduction of the relative expression levels of the three genes. The nuclear exosome degrades pre-mRNA and abnormally spliced mRNA in the nucleus [29,30]. The pre-mRNAs of several genes are degraded by the nuclear exosome in SSA-treated cells [27]. To investigate whether the nuclear exosome degrades the pre-mRNAs of the three genes of interest in SSA-treated cells, we knocked down *RRP4*, a component of the nuclear exosome, and confirmed successful knockdown using immunoblotting (Figure S2). We then measured the relative expression levels of exons in the three genes and found that the exons were not affected by the knockdown of *RRP4* (Figure 2E). Thus, the pre-mRNAs of the three genes were not degraded by the nuclear exosome.

We next assessed whether these pre-mRNAs are degraded by nonsense-mediated mRNA decay (NMD) [31,32]. To inhibit NMD, we simultaneously treated cells with SSA and cycloheximide (CHX) and then purified and analyzed newly transcribed mRNA. SSA decreased the expression level of three genes, but CHX did not suppress the downregulation of the genes (Figure 2F), indicating that these pre-mRNAs were not degraded by NMD.

### 2.3. Overexpression of CCNE1 and E2F1 Partially Suppresses G1 Phase Arrest

**Figure 3:**
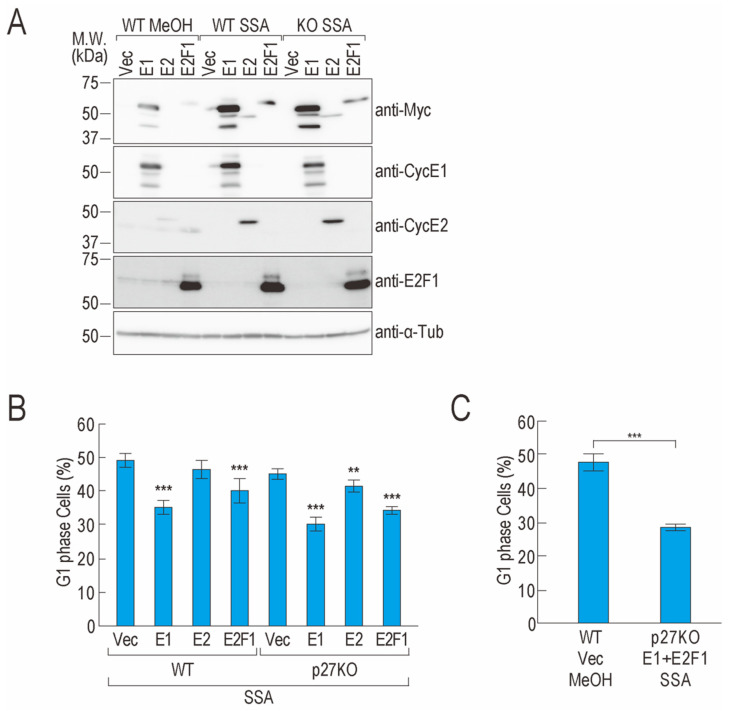
Overexpression of *CCNE1* and *E2F1* partially suppresses cell cycle arrest. (**A**,**B**) HeLa wild-type (WT) or p27 KO cells were transfected with pcDNA3.1-Myc/HIS (Vec), *CCNE1*-Myc (E1), *CCNE2*-Myc (E2), or *E2F1*-Myc (E2F1). The transfected cells were then treated with 10 ng/mL SSA or MeOH for 24 h. The proteins were analyzed using immunoblotting (**A**), and the cell cycle was analyzed using a cytometer (**B**). (**C**) HeLa WT cells or p27 KO cells were transfected with pcDNA3.1-Myc/HIS (Vec) or *CCNE1*-Myc (E1) and *E2F1*-Myc (E2F1). The transfected cells were treated with 10 ng/mL SSA or MeOH for 24 h. The cell cycle was analyzed using a cytometer. Error bars indicate standard deviation (n = 3). Statistical significance was investigated using one-way ANOVA and Dunnett’s test (**B**) or a two-tailed *t*-test (**C**) (** *p* < 0.01; *** *p* < 0.001).

SSA treatment decreased the protein levels of cyclin E1, cyclin E2, and E2F1 and caused G1 phase arrest. If a decrease in the levels of these three proteins causes G1 phase arrest following SSA treatment, overexpression of the three genes may suppress G1 phase arrest. To test this hypothesis, we constructed cyclin E1, cyclin E2, and E2F1 expression plasmids and confirmed protein overexpression; the expression level of cyclin E2 was lower than that of the other proteins (Figure 3A). To evaluate whether overexpression of the genes suppresses G1 phase arrest, we performed cell cycle synchronization using thymidine and plasmid transfection, after which we treated the cells with SSA. However, approximately 50% of the vector-transfected cells could not exit from the G1 phase, presumably because of transfection and thymidine treatment stress (unpublished data, D.K. and K.K.), which is consistent with our previous findings [23]. Therefore, we performed a similar experiment without cell cycle synchronization. As SSA treatment causes cell cycle arrest at G1 and G2/M phases [21,23], if overexpression of *CCNE1* and *E2F1* suppresses G1 phase arrest, the cells should be arrested at the next G2/M phase. Following SSA treatment, vector-transfected cells were arrested in both the G1 and G2/M phases, and overexpression of *CCNE1* and *E2F1* decreased the proportion of cells in the G1 phase and increased the proportion of cells in the G2/M phase (Figure 3B and Figure S3A,B). These results suggest that the overexpression of these two genes partially suppresses SSA treatment–induced G1 phase arrest.

Because these two genes did not suppress G1 phase arrest completely, we further investigated the effect of overexpression of the two genes in p27 knockout (KO) cells. We established p27KO cells using the CRISPR/Cas9 system and confirmed successful knockout using immunoblotting and sequencing (Figure S4). The proportion of p27KO cells with overexpression of *CCNE1* or *E2F1* in the G1 phase was smaller than that of wild-type cells with overexpression of *CCNE1* or *E2F1*, although the difference was not statistically significant (Figure 3B and Figure S3A,B; Table S1). Furthermore, we co-expressed *CCNE1* and *E2F1* in p27KO cells and treated the cells with SSA; however, the proportion of *CCNE1* and *E2F1* co-expressing cells in the G1 phase was similar to that of the *CCNE1* or *E2F1* single transfection groups (Figure 3B,C and Figure S3A–D). These results suggest that downregulation of *CCNE1* and *E2F1* expression contributes to G1 phase arrest in SSA-treated cells and other factors also contribute to the phenotype.

## 3. Discussion

In this study, we found that SSA treatment downregulated cyclin E1, cyclin E2, and E2F1 at both the protein and mRNA levels. However, Cdk2 protein was not affected by SSA treatment, at least under our experimental conditions. Thus, the following question arises: how does SSA treatment affect the gene expression of cell cycle regulators in a gene-specific manner? The protein and mRNA levels of cyclin E1, cyclin E2, and E2F1 oscillated in the untreated cells (Figure 1). We treated cells with SSA before these proteins started to accumulate. SSA treatment inhibited the new synthesis of these mRNAs and consequently inhibited the new synthesis of the proteins; therefore, we did not observe the expression of these genes in SSA-treated cells. However, because *CDK2* is expressed throughout the cell cycle (Figure 1), the level of Cdk2 protein, which was produced before SSA treatment, appeared to remain constant after SSA treatment. Therefore, the expression pattern of these cell cycle regulators and the timing of SSA treatment seem to be the causes of the downregulation of specific cell cycle regulators induced by SSA.

Our results showed that the mRNA and protein levels of cyclin E1, cyclin E2, and E2F1 were downregulated in SSA-treated cells. We investigated the reasons underlying the downregulation and found that transcription elongation of these three genes was defective following SSA treatment. We previously reported that the transcription elongation of ~20% of genes is inhibited in SSA-treated cells and that *CCNE1* and *CCNE2* are two genes with such transcription elongation defects [26,27]. In addition, we did find that the relative expression levels of exons 2–6 of *E2F1* were significantly lower than the levels of exon 1, suggesting that transcription elongation of *E2F1* is defective (Figure 2D). However, the relative expression level of the last exon of *E2F1* (*E2F1* exon 7) was higher than those of exons 2–6 (Figure 2D). Similarly, the relative expression levels of the last exons in *CCNE1* and *CCNE2* were higher than those of some of their upstream exons (*CCNE1* exons 10 and 11; *CCNE2* exons 7–10). This may be a technical bias of the exon array. To clarify this result, we will perform similar experiments using other methods such as next-generation sequencing. How transcription elongation is inhibited in splicing-deficient cells remains unclear. We previously demonstrated that some RNA-binding proteins suppress transcription elongation defects caused by SSA treatment [28]. These RNA-binding proteins might therefore be involved in the transcription elongation of G1 phase-related genes. We plan to evaluate the role of these RNA-binding proteins on the transcription elongation of G1 phase-related genes in a future study.

We also found that co-overexpression of *CCNE1* and *E2F1* combined with p27/p27* knockout did not completely suppress G1 arrest. We speculate that other G1 regulators may be downregulated in SSA-treated cells, leading to the SSA-induced G1 phase arrest. Numerous factors in addition to p27, cyclin E1, and E2F1 are involved in regulating the G1/S phase transition [3,9,24]. E2F1 activates the gene expression of numerous factors that drive the initiation of the S phase [7]. In *E2F1*-overexpressing cells, transcription initiation of genes important for the initiation of the S phase should be activated, but transcription elongation and splicing of the genes might be disrupted by SSA treatment. To understand the molecular mechanism underlying the G1 arrest caused by splicing inhibition comprehensively, future studies should investigate and identify the additional factors that contribute to G1 phase arrest.

In conclusion, we found that the gene expressions of *CCNE1*, *CCNE2*, and *E2F1* are downregulated in SSA-treated cells and that overexpression of *CCNE1* and *E2F1* suppresses SSA-induced G1 phase arrest. Splicing inhibitors such as SSA are potent antitumor reagents [21,33,34,35,36]. These findings, therefore, provide new insights into the molecular mechanism underlying the interconnection between splicing and cell cycle arrest and could potentially aid the development of novel cancer therapies based on splicing inhibitors.

## 4. Materials and Methods

### 4.1. Cell Culture, Synchronization, and Reagents

HeLa S3 cells were cultured in Dulbecco’s modified Eagle’s medium (FUJIFILM Wako Pure Chemical Corporation, Osaka, Japan) containing 10% heat-inactivated fetal bovine serum (Thermo Fisher Scientific, Waltham, MA, USA) at 37 °C with 5% CO_2_. For cell cycle synchronization, the cells were treated with 2 mM thymidine (FUJIFILM Wako Pure Chemical Corporation, Osaka, Japan) for 18 h. The treated cells were washed twice with medium to induce release from the first thymidine block and then cultured in fresh medium for 8 h. The cells were then treated with 2 mM thymidine for another 16 h and washed twice with culture medium to induce release from the double thymidine block. Cycloheximide was purchased from Sigma-Aldrich (St. Louis, MO, USA).

### 4.2. Antibodies and Immunoblotting

Mouse monoclonal anti-α-tubulin (B-5-1-2) antibody was purchased from Sigma-Aldrich (St. Louis, MO, USA). Mouse monoclonal anti-cyclin E1 (HE12), rabbit polyclonal anti-cyclin E2, rabbit polyclonal anti-E2F1, and rabbit monoclonal anti-p27 Kip1 (D69C12) antibodies were purchased from Cell Signaling Technology (Danvers, MA, USA). Mouse monoclonal anti-CDK2 antibody (D-12) was purchased from Santa Cruz Biotechnology (Dallas, TX, USA). Rabbit polyclonal anti-RRP4 antibody (ab156698) was purchased from Abcam (Cambridge, UK). Mouse monoclonal anti-Myc antibody was purchased from MBL (Nagoya, Japan). HRP-conjugated anti-mouse IgG and anti-rabbit IgG secondary antibodies were purchased from GE Healthcare (Little Chalfont, UK).

For immunoblotting, the cells were directly lysed on plates with 1× SDS-PAGE sample buffer. The cell lysates were then separated by SDS-PAGE and then transferred onto a PVDF membrane by electroblotting. Following incubation of the membrane with primary and secondary antibodies using standard techniques, protein bands were detected using the NOVEX ECL Chemiluminescent Substrate Reagent Kit (Thermo Fisher Scientific, Waltham, MA, USA) on an ImageQuant LAS 4000mini (GE Healthcare, Little Chalfont, UK).

### 4.3. Cell Cycle Analysis

The cells were fixed in 70% ethanol, rinsed with phosphate-buffered saline, and stained with a solution containing 20 µg/mL propidium iodide (Thermo Fisher Scientific), 0.05% Triton X-100, and 0.1 mg/mL RNase A (Thermo Fisher Scientific). Cell cycle progression was monitored using the image-based cytometer Tali (Thermo Fisher Scientific, Waltham, MA, USA).

### 4.4. siRNA Transfection

siGENOME Control Pool Non-Targeting #2 and siGENOME Human EXOSC2 siRNA for *RRP4* knockdown were purchased from Horizon Discovery (Cambridge, UK). siRNA transfection was performed using Lipofectamine RNAiMAX (Thermo Fisher Scientific, Waltham, MA, USA) in accordance with the manufacturer’s instructions.

### 4.5. RNA Purification and qRT-PCR

Total RNA was extracted from the cells using TRIzol reagent (Thermo Fisher Scientific, Waltham, MA, USA) in accordance with the manufacturer’s instructions. cDNA was prepared using Primescript II RTase (Takara, Otsu, Japan) and random primers. Nascent RNA was purified using the Click-iT Nascent RNA Capture Kit (Thermo Fisher Scientific, Waltham, MA, USA). Briefly, the cells were treated with 200 µM 5-EU for 1 h to label nascent RNAs. Total RNA was then extracted from the cells using TRIzol reagent. Labeled RNA was biotinylated via the click reaction and then purified using streptavidin beads. Subsequently, cDNA was synthesized using the SuperScript VILO cDNA Synthesis Kit (Thermo Fisher Scientific, Waltham, MA, USA). qRT-PCR was performed using the MX3000P system (Agilent, Santa Clara, CA, USA) with SYBR Green dye chemistry. The amount of 18S rRNA was measured as an internal control. All primers are listed in Table S2.

### 4.6. Plasmid Construction and Transfection

To construct the pcDNA3.1-GFP plasmid, the GFP gene was amplified by PCR from pcDNA6.2-enGFP (Thermo Fisher Scientific, Waltham, MA, USA) using GFP F-Hind III and GFP ATTTA-R-KpnI primers. The PCR product was digested with HindIII and KpnI and then subcloned into pcDNA3.1 (Thermo Fisher Scientific, Waltham, MA, USA). The DNA fragments of the *CCNE1*, *CCNE2*, and *E2F1* promoter regions were amplified by PCR from HeLa S3 genomic DNA using CCNE1 pro cloning for MluI and CCNE1 pro cloning rev HdIII primers, CCNE2 pro cloning for MluI and CCNE2 pro cloning rev HdIII primers, and E2F1 pro cloning for MluI and E2F1 pro cloning rev HdIII primers, respectively. The PCR products were digested with MluI and HindIII and subcloned into pcDNA3.1-GFP to construct pcDNA3.1-*CCNE1* promoter–GFP plasmid, pcDNA3.1-*CCNE2* promoter–GFP plasmid, and pcDNA3.1-*E2F1* promoter–GFP plasmid. To construct the *CCNE1*-Myc plasmid, the DNA fragment of *CCNE1* ORF was amplified by PCR from HeLa S3 cDNA using CCNE1 cloning for RI and CCNE1 cloning rev Xho primers. The PCR product was digested with EcoRI and XhoI and then subcloned into pcDNA3.1/Myc-HIS A (Thermo Fisher Scientific, Waltham, MA, USA). To construct the *CCNE2*-Myc plasmid, the DNA fragment of *CCNE2* ORF was amplified by PCR from HeLa S3 cDNA using CCNE2 cloning for RI and CCNE2 cloning rev Xho primers. The PCR product was digested with EcoRI and XhoI and then subcloned into pcDNA3.1/Myc-HIS A. To construct the *E2F1*-Myc plasmid, the DNA fragment of *E2F1* ORF was amplified by PCR from HeLa S3 cDNA using E2F1 cloning for RI and E2F1 cloning rev Xba primers. The PCR product was digested with EcoRI and XbaI and then subcloned into pcDNA3.1/Myc-HIS A. All primers are listed in Table S2. Plasmid transfection was performed using Lipofectamine 3000 reagent (Thermo Fisher Scientific, Waltham, MA, USA) in accordance with the manufacturer’s instructions.

### 4.7. Establishment of p27KO Cells

Alt-R^®^ CRISPR-Cas9 crRNA against *CDKN1B* (Design ID: Hs.Cas9.CDKN1B.1.AA), Alt-R^®^ CRISPR-Cas9 tracrRNA, and Alt-R^®^ S.p. HiFi Cas9 Nuclease V3 were purchased from Integrated DNA Technologies (Coralville, IA, USA). Formation of guide RNA (crRNA:tracrRNA duplex), formation of the Cas9–guide RNA complex, and electroporation using the Neon transfection system (Thermo Fisher Scientific, Waltham, MA, USA) were each performed in accordance with the manufacturer’s instructions. Clone selection and validation via immunoblotting and sequencing were then performed. All primers are listed in Table S2.

### 4.8. Exon Array Data Analysis

Probeset intensities of the core probe sets, as defined by Affymetrix, were calculated from the CEL files, which were previously deposited in the GEO database (https://www.ncbi.nlm.nih.gov/geo/query/acc.cgi?acc=GSE45379) (accessed on 26 October 2021) [27], using Partek Genomic Suite 6.5 (Partek, St. Louis, MO, USA) with default settings at the probeset level. Probesets with low expression levels (mean < 3) and high statistical dispersion (standard deviation > 10% of the mean) were excluded.

### 4.9. Statistical Analysis

Statistical analysis was performed using R Commander. A two-tailed *t*-test (Figure 1C, Figure 2B and Figure 3C) and one-way ANOVA with Tukey’s test (Figure 2C,E,F and Figure 3B) or Dunnett’s test (Figure 2D and Figure 3B) were performed to determine statistical significance. Data are presented as means ± standard deviation. The sample size used in each experiment is stated in the figure legends. *p* < 0.05 was considered statistically significant.

## Supplementary Materials

The following are available online at https://www.mdpi.com/article/10.3390/ijms222111623/s1.
