# Supplementary material for: CCNE1 and E2F1 Partially Suppress G1 Phase Arrest Caused by Spliceostatin A Treatment
Source: Int J Mol Sci. 2021 Oct 27;22(21):11623. doi: 10.3390/ijms222111623 (PMC8584075; doi:10.3390/ijms222111623)
Supplement: Supplementary file 1 [file ijms-22-11623-s001.zip › ijms-1414137-supplementary.pdf]

Supplementary Information

Cyclin E1 and E2F1 partially suppress G1 phase arrest caused by spliceostatin A treatment

Kei Kikuchi and Daisuke Kaida*

Faculty of Medicine, Academic Assembly, University of Toyama, Toyama, 930-0194, Japan

*To whom correspondence should be addressed.

Tel./Fax: +81-76-415-8848

E-mail: kaida@med.u-toyama.ac.jp

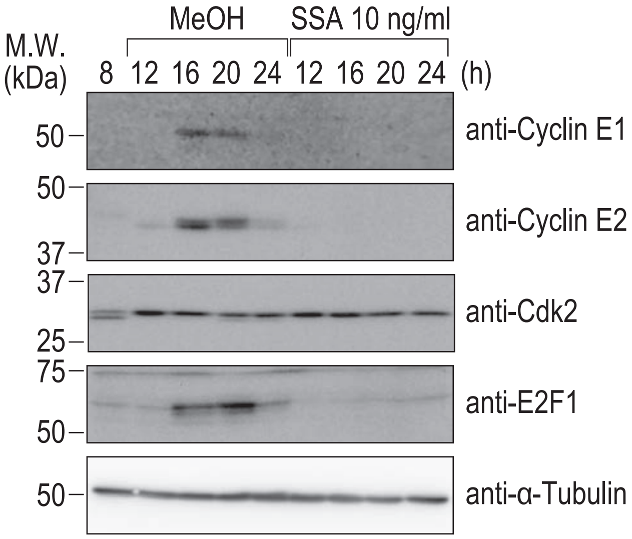

Figure S1. SSA treatment decreases the protein levels of cell cycle regulators

Eight hours after release from a double thymidine block, synchronized HeLa S3 cells were treated with MeOH or 10 ng/ml SSA. The cells were then harvested at the indicated time points. The protein levels of cell cycle regulators were analyzed using immunoblotting. Molecular weights are indicated to the left of the gels; antibodies are indicated to the right of the gels. The protein level of α-tubulin was measured as an internal control.

Fig. S1 Kikuchi and Kaida

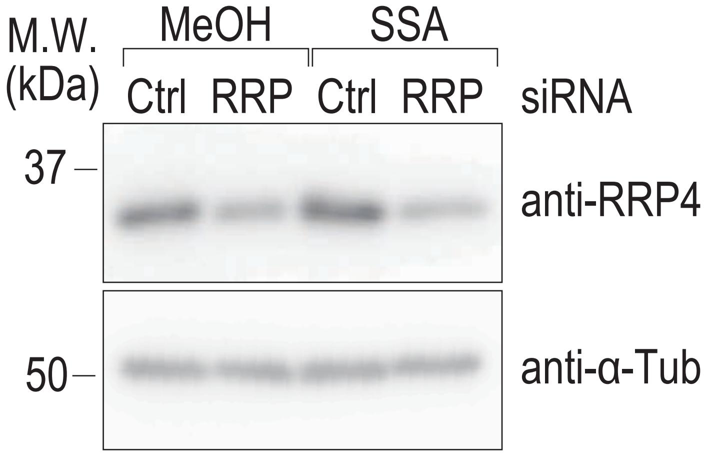

Figure S2. Confirmation of successful knockdown of *RRP4*

Synchronized cells were released from the first thymidine block and transfected with *RRP4* siRNA (RRP) or control siRNA (Ctrl). Eight hours after release from the second thymidine block, the cells were treated with 10 ng/ml of SSA or MeOH for 4 hours, then analyzed by immunoblotting.

Fig. S2 Kikuchi and Kaida

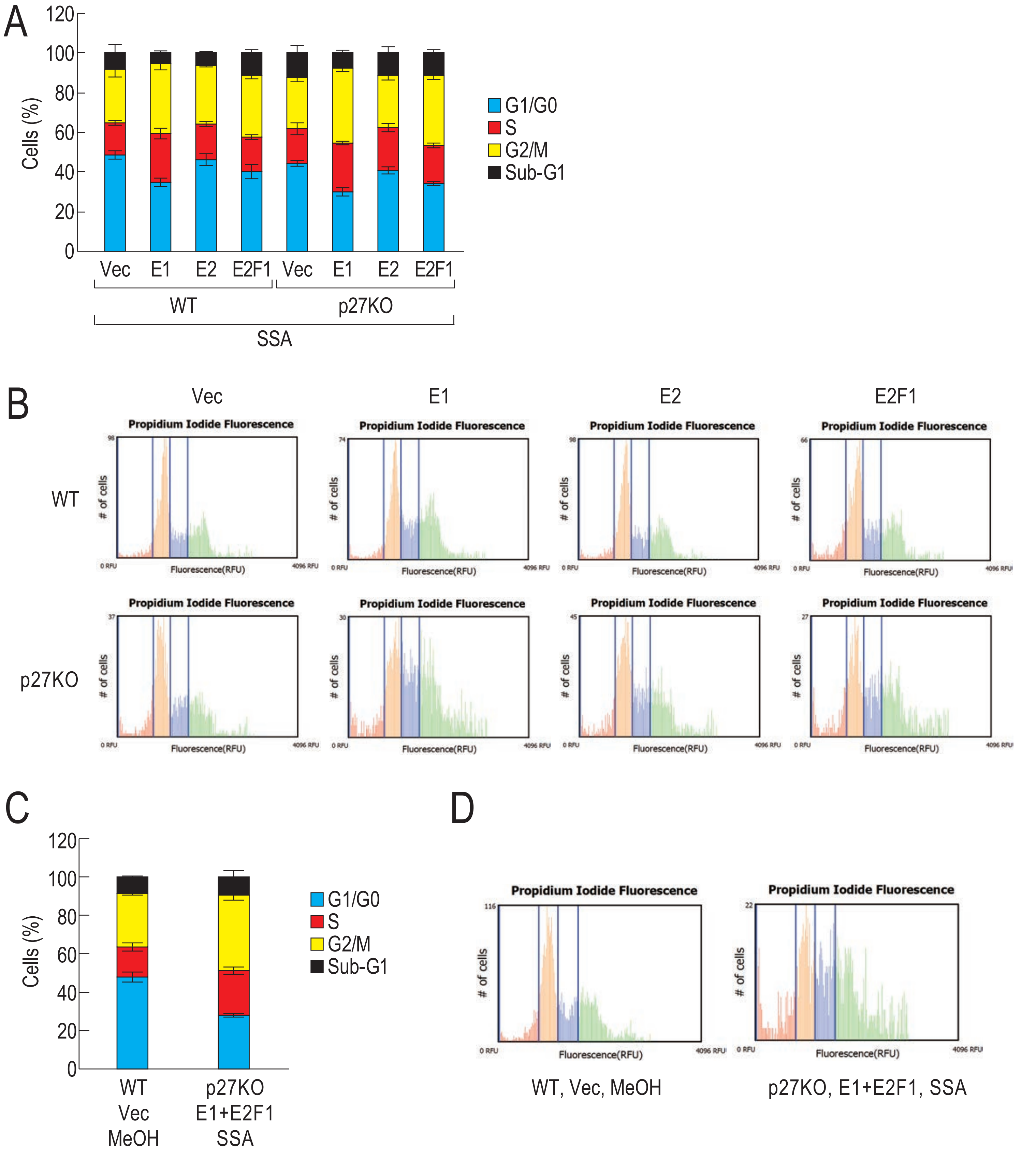

Figure S3. Overexpression of *CCNE1* and *E2F1* partially suppresses G1 phase arrest caused by splicing inhibition

(A) HeLa cells or p27 KO cells were transfected with pcDNA3.1-Myc/HIS (Vec), CCNE1-Myc (E1), CCNE2-Myc (E2) or E2F1-Myc (E2F1). The transfected cells were treated with 10 ng/ml of SSA for 24 h. Cell cycle of the cells was analyzed by a cytometer. Error bars indicate standard deviation (n = 3).

(B) Representative histograms from (A).

(C) HeLa cells or p27 KO cells were transfected with pcDNA3.1-Myc/HIS (Vec) or CCNE1-Myc (E1) and E2F1-Myc (E2F1). The transfected cells were treated with 10 ng/ml SSA or MeOH for 24 h. The cell cycle was analyzed using a cytometer. Error bars indicate standard deviation (n = 3).

(D) Representative histograms from (C).

Fig. S3 Kikuchi and Kaida

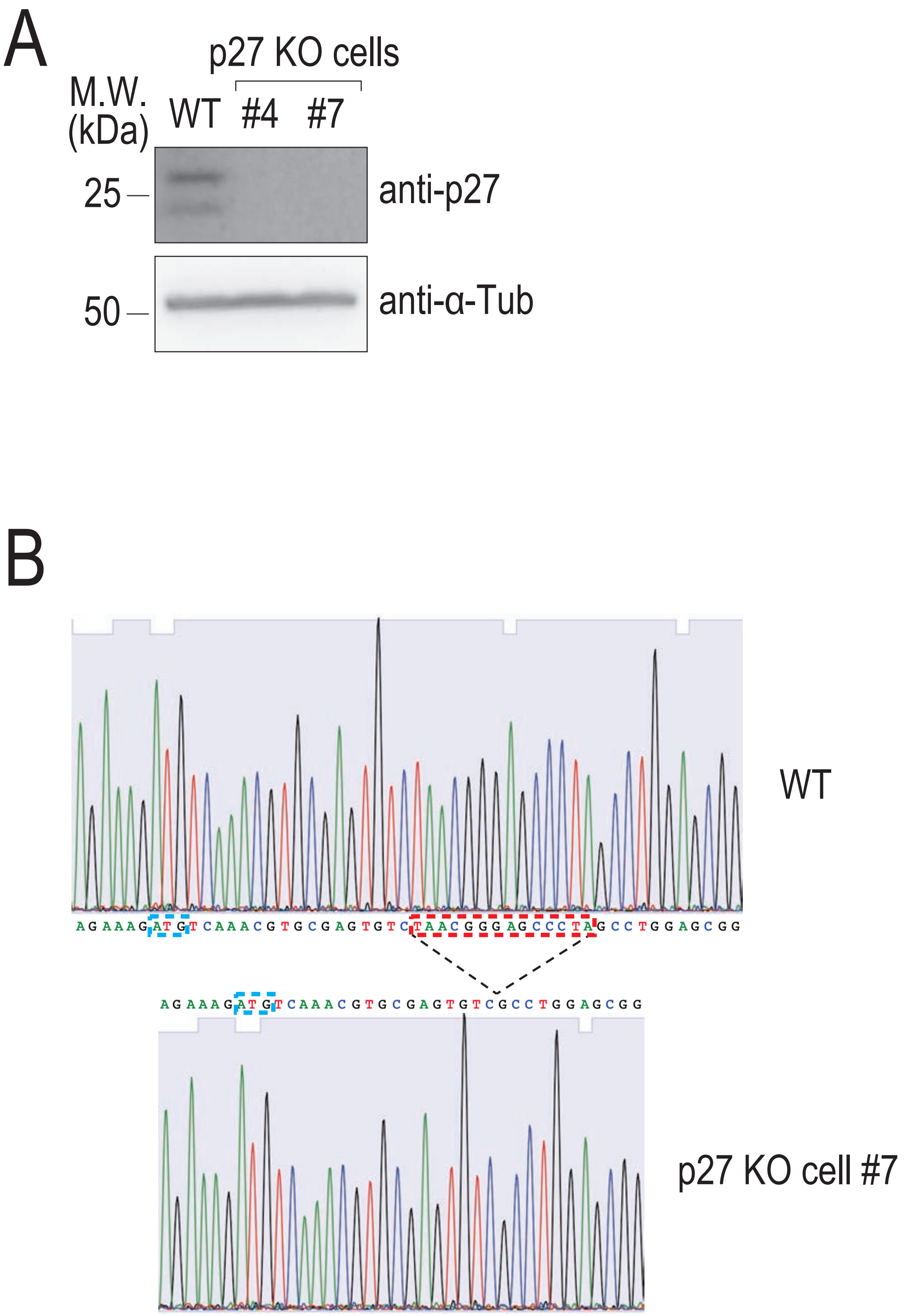

Figure S4. Confirmation of successful knockout of CDKN1B

(A) HeLa S3 cells and p27KO cells (clone #4 and #7) were analyzed by immunoblotting. Molecular weights are indicated to the left of the gels. (B) DNA sequences of HeLa S3 cells and p27KO cells. Cyan dashed rectangles indicate the start codon. A red dashed rectangle indicates the deleted 13 nt in the p27KO cells.

Table S1. values of samples from Fig. 3B, as calculated using Tukey's test

Sample1	Sample2	p-value
WT Vec	WT CycE1	0.0000201
WT Vec	WT CycE2	0.8134967
WT Vec	WT E2F1	0.0028149
WT Vec	p27KO Vec	0.3114226
WT Vec	p27KO CycE1	0.0000003
WT Vec	p27KO CycE2	0.0082047
WT Vec	p27KO E2F1	0.0000083
WT CycE1	WT CycE2	0.0002523
WT CycE1	WT E2F1	0.176808
WT CycE1	p27KO Vec	0.0013917
WT CycE1	p27KO CycE1	0.176808
WT CycE1	p27KO CycE2	0.0675322
WT CycE1	p27KO E2F1	0.9990611
WT CycE2	WT E2F1	0.048043
WT CycE2	p27KO Vec	0.9798145
WT CycE2	p27KO CycE1	0.0000027
WT CycE2	p27KO CycE2	0.1298636
WT CycE2	p27KO E2F1	0.0000947
WT E2F1	p27KO Vec	0.2369103
WT E2F1	p27KO CycE1	0.0009825
WT E2F1	p27KO CycE2	0.9990611
WT E2F1	p27KO E2F1	0.0675322
p27KO Vec	p27KO CycE1	0.0000111
p27KO Vec	p27KO CycE2	0.5010504
p27KO Vec	p27KO E2F1	0.0004944
p27KO CycE1	p27KO CycE2	0.0003526
p27KO CycE1	p27KO E2F1	0.4002237
p27KO CycE2	p27KO E2F1	0.0238961

Table S2. List of primers used in this study.

For cloning	
Name	Sequence
GFP F-Hind III	CGCAAGCTTAACATGGTGAGCAAGGGCGA
GFP ATTTA-R-KpnI	GCGGTACCTAAATTAAATTAAATTAAATTAAATTAAATT TACTTGTACAGCTCGTCCATG
CCNE1 pro cloning for MluI	GCCGCCACGCGTGAATGGACAGGCGGCCAGGAATAG
CCNE1 pro cloning rev HdIII	GGCGGCAAGCTTCTTCATGGTGTCCCGCTCCTTCG
CCNE2 pro cloning for MluI	GCCGCCACGCGTGAAGAGAGGAAGCAAGGGAG
CCNE2 pro cloning rev HdIII	GGCGGCAAGCTTGTCTAGTTCTCAGCCCTCCC
E2F1 pro cloning for MluI	GCCGCCACGCGTCTCGGGCTCAAGCAATCCTC
E2F1 pro cloning rev HdIII	GGCGGCAAGCTTCGCGCCAAATCCTTTTTGCC
CCNE1 cloning for RI	GCCGCCGAATTCATCATGCCGAGGGAGCG
CCNE1 cloning rev Xho	GGCGGCCTCGAGCGCCATTTCCGGCCC
CCNE2 cloning for RI	GCCGCCGAATTCGAGAATGTCAAGACGAAGTAGCCG
CCNE2 cloning rev Xho	GGCGGCCTCGAGGTGTTTTCCTGGTGGTTTTTCAGTG
E2F1 cloning for RI	GCCGCCGAATTCGTCATGGCCTTGGCC
E2F1 cloning rev Xba	GGCGGCTCTAGAGAAATCCAGGGGGGTGAG
For RT-qPCR	
Name	Sequence
18S rRNA for	GTTGGTGGAGCGATTTGTCTGGTT
18S rRNA rev	TATTGCTCAATCTCGGGTGGCTGA
CCNE1 Ex3 for	GAAGGAGCGGGACACCATGAAG
CCNE1 Ex3 rev	GGTCACGTTTGCCTTCCTCTTCC
CCNE1 Ex6 for	GGGCAAATAGAGAGGAAGTCTG
CCNE1 Ex6 rev	AGGGTGTTGCTCAAGAAAGT
CCNE1 Ex6-7 for	CTTGAGCAACACCCTCTTCT
CCNE1 Ex6-7 rev	AAAGGTCTCCCTGTGAAGTTTAT
CCNE1 Ex7 for	GGTATATGGCGACACAAGAAA
CCNE1 Ex7 rev	CTCAAGTTTGGCTGCAATAAAT
CCNE2 Ex5 for	GGGATCAGTCCTTGCATTATCA
CCNE2 Ex5 rev	ATCAGGCAAAGGTGAAGGATTA
CCNE2 Ex11 for	TTGGAGTGGGACAGTATTTCAG
CCNE2 Ex11 rev	AAGTCTTCAGCTTCACTGGAC
CCNE2 Ex11-12 for	GGCTATGCTGGAGGAAGTAAAT
CCNE2 Ex11-12 rev	GCTCTTCGGTGGTGTCATAAT
CCNE2 Ex12 for	CCACCGAAGAGCACTGAAA
CCNE2 Ex12 rev	CAGTGATACCAGTTCTACCCAATC
E2F1 Ex3 for	CGCTATGAGACCTCACTGAATC
E2F1 Ex3 rev	GGACGTTGGTGATGTCATAGAT
E2F1 Ex6 for	TCACTTCTGAGGAGGAGAACA
E2F1 Ex6 rev	TAGAGACTGGCTGGGATCTG
E2F1 Ex6-7 for	GTCACCACCACCATCATCTC

E2F1 Ex6-7 rev	ACAACAGCGGTTCTTGCT
E2F1 Ex7 for	AGGAGTTCATCAGCCTTTCC
E2F1 Ex7 rev	CCCAAAGTCACAGTCGAAGA
GFP-2 for	CTTCTTCAAGTCCGCCATGC
GFP-2 rev	CTTCAGCTCGATGCGGTTCA
For knockout check	
Name	Sequence
p27 KO check F	CGCTCGCCAGTCCATTT
p27 KO check R	CATGTCTCTGCAGTGCTTCT
